# Efficacy and safety of Keluoxin capsule in combination with Western medicine for diabetic kidney disease: A systematic review and meta-analysis

**DOI:** 10.3389/fphar.2022.1052852

**Published:** 2023-01-04

**Authors:** Wenhua Zhang, Jingxin Zhou, Churan Wang, Xu Wang, Shuwen Zhang, Weiyu Jia, Yijia Jiang, Lan Lin, Yanbing Gong

**Affiliations:** ^1^ Dongzhimen Hospital, Beijing University of Chinese Medicine, Beijing, China; ^2^ Guang’anmen Hospital China Academy of Chinese Medicine Sciences, Beijing, China

**Keywords:** Keluoxin capsule, diabetic kidney disease, systematic review, meta-analysis, Chinese patent medicine

## Abstract

**Objective:** Keluoxin capsule (KLXC) has been widely used in diabetic kidney disease (DKD), but its efficacy and safety have not yet been clarified. A systematic review and meta-analysis were performed to assess the efficacy and safety of KLXC for DKD.

**Methods:** The randomized control trials (RCTs) included KLXC searched from seven major English and Chinese databases up until 3 June 2022. The methodological quality and risk of bias were assessed by version 2 of the Cochrane risk-of-bias tool (RoB 2) for the RCTs from the Cochrane Handbook. The analyses were conducted by RevMan 5.4 and Stata 17.0.

**Results:** A total of 20 trials with 1,500 participants were identified. The meta-analysis showed that KLXC combined with Western medicine was superior to the use of Western medicine alone for DKD which included improvements in the estimated glomerular filtration rate (eGFR) [MD = 3.04, 95% CI (0.30, 5.78), *p* = 0.03], reduction in microalbuminuria (mALB) [MD = −25.83, 95% CI (−41.20, −10.47), *p* = 0.001], urinary albumin excretion rate (UAER) [SMD = −0.97, 95% CI (−1.50, −0.44), *p* = 0.0003], 24-h urine protein (24hUpro) [SMD = −1.31, 95% CI (−1.82, −0.80), *p* < 0.00001], serum creatinine (Scr) [MD = −11.39, 95% CI (−18.76, −4.02), *p* = 0.002], blood urea nitrogen (BUN) [MD = −1.28, 95% CI (−1.67, −0.88), *p* < 0.00001], fasting blood glucose (FBG) [MD = −0.51, 95% CI (−0.90, −0.11), *p* = 0.01], total cholesterol (TC) [MD = −1.04, 95% CI (−1.40, −0.68), *p* < 0.00001], triglycerides (TG) [MD = −0.36, 95% CI (−0.50, −0.23), *p* < 0.00001], and low-density lipoprotein cholesterol (LDL) [MD = −0.39, 95% CI (−0.71, −0.07), *p* = 0.02]. Results showed no statistically significant difference in glycated hemoglobin (HbA1c) (*p* = 0.14) or adverse events (*p* = 0.81) between the two groups.

**Conclusion:** The combination of KLXC and Western medicine had a positive effect on DKD. However, due to the high clinical heterogeneity and low quality of included studies, further standardized, large-scale, rigorously designed RCTs for DKD in the definitive stage are still necessary to achieve more accurate results.

**Systematic Review Registration:** https://inplasy.com/inplasy-2021-11-0067/, identifier INPLASY 2021110067.

## 1 Introduction

Diabetic kidney disease (DKD), as one of the most serious microvascular complications of diabetes mellitus (DM), is characterized by persistent albuminuria or reduced estimated glomerular filtration rate (eGFR) due to chronic exposure to hyperglycemia, resulting in progressive alterations in the kidney structure and function (Alicic et al., 2017; Selby and Taal, 2020). Epidemiological studies have shown that approximately 20%–50% of DM developed into DKD, of which 50% progressed to end-stage renal disease (ESRD) requiring dialysis or kidney transplantation with an increased risk of cardiovascular disease and premature mortality (Afkarian et al., 2013; Selby and Taal, 2020). The improvement of health outcomes should refocus on the strategies to control the progression of DKD to ESRD.

Improving albuminuria and reducing eGFR in DKD patients are important treatments to delay the progress to ESRD (Banerjee et al., 2021). Current therapies to postpone the progression of CKD include angiotensin-converting enzyme inhibitors (ACEIs), angiotensin II receptor blockers (ARBs), and sodium–glucose cotransporter 2 (SGLT2) inhibitors (Barrera-Chimal et al., 2021; Wang et al., 2021; Ravindran and Munusamy, 2022). Moreover, the coexistence of multiple risk factors and concurrent comorbidities leads to an increasing number of combined medications, while reduced drug clearance and consequent side effects also limit the choice of treatments (Tuttle et al., 2014). Therefore, the search for additional complementary and alternative combination therapies remains urgently necessary for DKD.

Chinese medicine possessed promising clinical benefits as primary or alternative therapies for DKD with rarely observed adverse effects (Tang et al., 2021). KLXC is a proprietary Chinese medicine widely used in China for DKD. It is composed of *Astragalus membranaceus*, glossy privet fruit, leech, Rheum officinale, Pseudostellaria heterophylla, and Lycii fructus. The overall ingredients of KLXC are listed in Table 1. KLXC has the function of tonifying Qi and Yin and activating the blood circulation to remove blood stasis (Endocrine Professional Committee of the Chinese Society of Integrative Medicine, 2020). Previous clinical studies have demonstrated that KLXC exerts therapeutic effects on DKD by improving glucolipid metabolism, regulating microcirculation, and preventing kidney damage (Lin et al., 2000). KLXC is recommended as a Class 1A proprietary Chinese medicine in the Chinese DKD guidelines (Yu et al., 2022), increasing frequent RCTs on KLXC supplemental therapy for DKD were reported in China. These RCTs have not only demonstrated positive effects of KLXC on glucolipid metabolism but also contributed to improved renal function with mild manageable adverse effects (Zhao et al., 2017; Bai et al., 2019; Yang et al., 2021). However, systematic evaluations of its efficacy and safety for DKD are scarce. Therefore, a systematic review and meta-analysis were conducted to comprehensively evaluate the benefits and drawbacks of KLXC for DKD to provide trustworthy evidence for clinical applications.

**TABLE 1:** Complete name and species of ingredients of KLXC.

Chinese name	Pharmaceutical name	Species	Family
Huangqi	Astragalus membranaceus	*Astragalus membranaceus* (Fisch.) Bge.	Leguminosae
Nvzhenzi	Glossy privet fruit	*Ligustrum lucidum* Ait.	Myrtaceae
Shuizhi	Leech	*Antiaris toxicaria* (Pers.) Lesch.	Leechidae
Dahuang	Rheum officinale	*Rheum officinale* Baill.	Polygonaceae
Taizishen	Pseudostellaria heterophylla	*Pseudostellaria heterophylla* (Miq.) Pax	Caryophyllaceae
Gouqizi	Lycii fructus	*Lycium barbarum* L.	Solanaceae

## 2 Methods

### 2.1 Study registration

The protocol for this study had been registered on INPLASY (ID: INPLASY 2021110067) and conducted on the basis of the Preferred Reporting Items for Systematic Reviews and Meta-Analysis Protocol statement guidelines (Shamseer et al., 2015). As all the research materials required were published studies, no ethical approval was required for conducting this study.

### 2.2 Inclusion and exclusion criteria

#### 2.2.1 Type of studies

Only RCTs were eligible for inclusion, regardless of the languages. Animal experiments, case reports, nonclinical research, commentaries, and repeated publications were not included. RCTs with incomplete and unavailable important data were excluded.

#### 2.2.2 Type of participants

The study included adult participants aged 18 years or older who were diagnosed with DKD according to the Kidney Disease Outcomes Quality Initiative (KDOQI) criteria (National Kidney Foundation, 2012). There were no restrictions on the type of DM, stage of DKD, gender, nationality, race, education, or job.

#### 2.2.3 Type of interventions

We considered all intervention trials that met the inclusion criteria, which included treatment with KLXC on an unrestricted dosage or course. Comparators consisted of any Western medicine, placebo, or no intervention. Both groups received the same conventional treatments for DKD, which included comprehensive management of glycemia, blood pressure, serum lipid level, lifestyle, and nutrition following the recommendations of the KDOQI clinical practice guidelines. Any herbal or Chinese medicine treatments were excluded from the analysis.

#### 2.2.4 Primary and secondary outcomes

The primary outcomes included eGFR, microalbuminuria (mALB), urinary albumin excretion rate (UAER), and 24-h urine protein (24hUpro). The secondary outcomes included kidney function (Scr, BUN), glucose (FBG, HbA1c), and lipids (TC, TG, and LDL). Additional outcomes were adverse events.

### 2.3 Search strategy

The China National Knowledge Infrastructure (CNKI), Wanfang data (WF Data), VIP database, SinoMed, PubMed, Web of Science (WOS), and Cochrane Library (Clib) were searched to ensure all possible RCTs on KLXC for DKD, without language restrictions. The time interval for literature searching was from the inception of the libraries to 3 June 2022. The key search terms were “diabetic kidney disease” or “diabetic nephropathy,” “keluoxin,” and “random.” The search strategies are shown in Supplementary Table S1.

### 2.4 Study selection

Records were extracted from each database and imported into EndNote 20, and duplicates were removed. Two independent reviewers screened the titles and abstracts to exclude irrelevant studies and reviewed full texts according to the inclusion and exclusion criteria. Any disagreements between the two reviewers were resolved by a third reviewer. Additionally, references to the included studies were searched for further potentially relevant articles. If the same clinical data were published more than once, the report that contained the most recent and comprehensive information such as the largest sample size was included.

### 2.5 Data extraction and analysis

#### 2.5.1 Data extraction

Two reviewers independently extracted the following information from the selected studies with a predefined form: first author, year of publication, country, stage of DKD, study design, sample size, gender, age, dose and course of KLXC, type of control, trial cycle, and outcomes. If information was missing or unavailable directly from the articles, we contacted the corresponding authors to obtain the data and documented all contacts. Studies were excluded if we were unable to obtain the relevant data.

#### 2.5.2 Risk of bias assessment

The methodological quality and risk of bias of the included studies were assessed independently by two reviewers using version 2 of the Cochrane risk-of-bias tool (RoB 2) of the Cochrane Handbook for RCTs (Cumpston et al., 2022). Sources of bias assessment included the randomization process, deviations from the intended intervention, missing outcome data, measurement of the outcome, and selection of the reported result. Any disagreements were resolved by a third reviewer.

#### 2.5.3 Data synthesis and analysis

Statistical analyses were performed using the RevMan software (version 5.4, Copenhagen: The Nordic Cochrane Center, The Cochrane Collaboration). Continuous variables that included the primary and secondary outcomes were evaluated by standardized mean differences (SMD) or mean differences (MD) with 95% confidence intervals (95% CIs). Dichotomous outcomes were measured by odds ratios (ORs) and their 95% CIs (Bakbergenuly et al., 2019). An OR < 1.00 means that exposure to the risk variable reduces the risk of the event. An OR > 1.00 means that the risk is increased. The statistical significance of an OR is stated along with the OR and its 95% CI. If the 95% CI for the OR includes 1.00, the OR is not statistically significant (Andrade, 2015).

Heterogeneity among studies was assessed by the *I* ^2^ test (Higgins and Thompson, 2002), and the data with low heterogeneity (*I* ^2^ < 50%, *p* ≥ 0.1) were assessed as a fixed effects model. Data with significant heterogeneity (*I* ^2^ > 50%, *p* < 0.1) were assessed as a random effects model for meta-analysis. If the data could not be meta-analyzed, descriptive analysis was applied.

#### 2.5.4 Subgroup and sensitivity analysis

If there was high heterogeneity and sufficient data, the subgroup analysis was performed to explore the possible sources of high heterogeneity between studies based on the stage of DKD, age, trial cycle, etc. If heterogeneity was high but data were small, sensitivity analysis was performed by removing each study individually and observing its impact on the overall results.

#### 2.5.5 Publication bias assessment

Outcomes reported more than 10 times (Sterne et al., 2011) were selected to assess publication bias by Egger’s test using Stata 16.0, and studies were considered to have publication bias if *p* < 0.05.

## 3 Results

### 3.1 Search results

**FIGURE 1:**
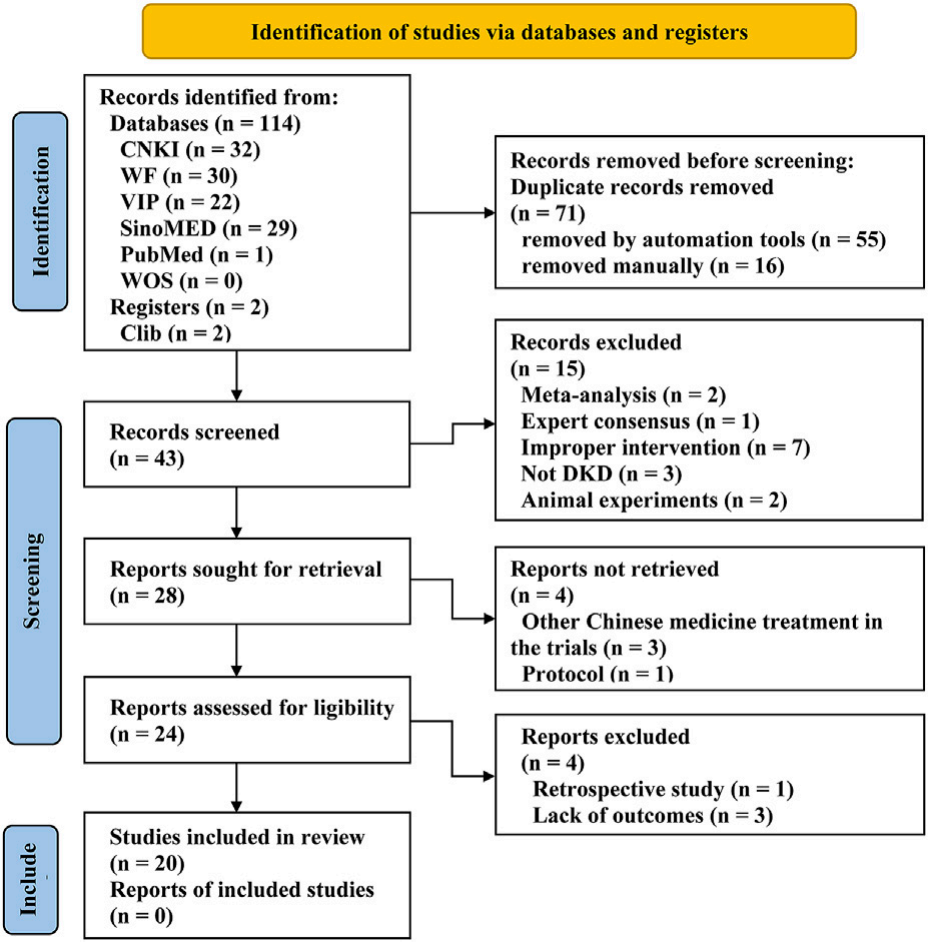
Flow diagram of the search for eligible studies.

A total of 114 articles were retrieved according to the search queries. After removing 71 duplicates by EndNote and manually, 43 articles remained for further examination. After scanning the titles and abstracts, 19 studies were removed. After reading the full texts, 4 articles were excluded for the ineligible study type (*n* = 1) and missing outcomes (*n* = 3), and ultimately 20 qualified trials (He et al., 2012; Hou, 2012; Liu et al., 2012; Hu et al., 2013; Ju et al., 2013; Zhou, 2013; Chen, 2014; He et al., 2014; Shen, 2015; Wei et al., 2015; Li et al., 2016; Wang, 2016; Zhang, 2016; Guo et al., 2019; Hou, 2019; Chen et al., 2020; Fu et al., 2020; Jin, 2020; Cui, 2021; Yu and Liang, 2021) were identified for further systematic review and meta-analysis. The overall screening process is detailed in the PRISMA (Page et al., 2021) flow diagram (Figure 1).

A total of 20 trials with 1,500 DKD patients (treatment group, 755; control group 745) were included with the intervention duration ranging from 4 weeks to 6 months, and all of the studies were conducted and published in China. There were 18 trials (Liu et al., 2012; Hu et al., 2013; Ju et al., 2013; Zhou, 2013; Chen, 2014; Shen, 2015; Wei et al., 2015; Li et al., 2016; Wang, 2016; Hou, 2019; Chen et al., 2020; Jin, 2020; Cui, 2021; Yu and Liang, 2021) treated with the combination of KLXC and Western medicine in the experiment group, and Western medicine alone in the control group. There was one trial (Hou, 2012) conducted with conventional treatment in the control group and supplemented with KLXC in the experiment group. There was one trial (Zhang, 2016) that had KLXC in the treatment group and KLXC placebo in the control group in addition to Western medicine treatment. The specific characteristics of the included 20 trials are displayed in Table 2.

**TABLE 2:** The characteristics of the included trials.

Study ID	Stage of DKD	Sample Size (male)		Age (rang/mean, year)	Interventions		Trial Cycle	Indictors			
					T	C		Kidney	glucose	Lipid	Safety
He et al, (2012)	DN III	T:22 C:23	T:42(22) C:44(21)	T:53.5 ± 8.7 C:55.1 ± 9.1	KLX 2 g tid + Candesartan cilexetil 8 mg qd	Candesartan cilexetil 8 mg qd	2 m	②⑤⑥	⑩	⑬⑭⑮⑯	
	DN IV	T:21 C:20						②⑤⑦			
Hou (2012)	DN III DN IV	T:25(12) C:26(17)		T:61.76 ± 9.99 C:62.20 ± 10.96	KLX 2 g tid + Conventional treatments	Conventional treatments	8 w	①②④⑦⑨	⑫		⑰⑱⑲
Liu et al. (2012)	DN III	T:21 C:22	T:39(21) C:41(20)	T:53.5 ± 8.7 C:55.1 ± 9.1	KLX 2 g tid + Enalapril 10 mg qd	Enalapril 10 mg qd	2 m	②⑤⑥	⑩	⑬⑭⑮⑯	
	DN IV	T:18 C:19						②⑤⑦			
Hu et al. (2013)	DN IV	T:32(20) C:28(11)		T:54.71 ± 9.5 C:54.46 ± 9.6	KLX 2 g tid + Valsartan 80 mg qd	Valsartan 80 mg qd	8 w	②⑤⑦			⑰⑱
Ju et al. (2013)	DN III	T:25(14) C:25(13)		T:51.2 ± 6.4 C:52.6 ± 6.2	KLX 2 g tid + Benazepril 10 mg qd	Benazepril 10 mg qd	8 w	②③⑨	⑩⑪⑫	⑬⑭⑮	⑰⑱⑲⑳
Zhou (2013)	DN III	T:54 C:48		T:57.48 ± 6.78 C:58.2 ± 7.16	KLX 2 g tid + Irbesartan 150 mg qd	Irbesartan 150 mg qd	8 w	②⑤⑦			⑰⑱
He et al. (2014)	Early DN	T:22(13) C:23(12)		T:55.3 ± 9.5 C:53.1 ± 8.7	KLX 2 g tid + Candesartan cilexetil 8 mg qd	Candesartan cilexetil 8 mg qd	2 m	②⑥	⑩		⑰
Chen (2014)	DN III DN IV	T:30 C:28		36 ± 22.5	KLX 2 g tid + Alprostadil 10 μg qd	Alprostadil 10 μg qd	15 d	①⑥	⑩	⑬⑭⑮⑯	⑰
Shen (2015)	DN III DN IV	T:40(21) C:40(18)		T:52.7 ± 9.0 C:54.1 ± 8.6	KLX 2 g tid + Candesartan cilexetil 8 mg qd	Candesartan cilexetil 8 mg qd	2 m	②⑤	⑩	⑬⑭⑮⑯	
Wei et al. (2015)	DN III DN IV	T:42(24) C:40		T:37 ± 21.5 C:36.5 ± 20.7	KLX 2 g tid + Alprostadil 10 μg qd	Alprostadil 10 μg qd	4 w	①②③⑧	⑩		
Li et al. (2016)	DN	T:53(30) C:53(32)		T:61.34 ± 10.83 C:63.03 ± 9.16	KLX 2 g tid + Telmisartan 80 mg qd	Telmisartan 80 mg qd	8 w	①⑥⑦		⑬⑭	⑰
Wang (2016)	DN III DN IV	T:50(30) C:50(31)		T:64.5 C:64.2	KLX 2 g tid + Alprostadil 10 μg qd	Alprostadil 10 μg qd	4 w	①②③⑧	⑩		⑱
Zhang (2016)	Early DN	T:75(39) C:74(40)		T:47.4 ± 5.2 C:47.3 ± 5.4	KLX 2 g tid + Irbesartan 150 mg qd	KLX placebo + Irbesartan 150 mg qd	24 w	①②③⑥⑦	⑩⑪	⑬⑭⑮⑯	⑰⑱⑳
Guo et al. (2019)	DN III DN IV	T:30(16) C:30(17)		T:55.69±6.32 C:55.83±6.42	KLX 2 g tid + Compound α-ketoacid tablet 2.52 g tid	Compound α-ketoacid tablet 2.52 g tid	3 m	①②③⑦			⑰
Hou (2019)	DN	T:48(26) C:48(28)		T:53.01 ± 4.87 C:52.18 ± 5.04	KLX 2 g tid + Benazepril 10 mg qd	Benazepril 10 mg qd	1 m	②③	⑩⑪⑫		
Chen et al. (2020)	DN	T:30(16) C:30(17)		T:53.80 ± 6.17 C:53.68 ± 6.09	KLX 2 g tid + Benazepril 10 mg qd	Benazepril 10 mg qd	2 m	①②③⑥⑦			
Fu et al. (2020)	DN	T:43 C:43		38 ± 20.8	KLX 2 g tid + Olmesartan medoxomil 20 mg qd	Olmesartan medoxomil 20 mg qd	6 m	①②③⑧	⑩		
Jin (2020)	DN III	40	T:40(22) C:40(23)	T:53.38 ± 5.66 C:53.43 ± 5.51	KLX 2 g tid + Ramipril 5 mg qd	Ramipril 5 mg qd	8 w	②⑤⑥	⑩		⑰
	DN IV	40						②⑤⑦			
Cui (2021)	DN III	T:31 C:32	T:57(32) C:56(30)	T:59.02 ± 5.28 C:58.75 ± 5.32	KLX 2 g tid + Benazepril 10 mg qd	Benazepril 10 mg qd	4 w	①②③⑥			
	DN IV	T:26 C:24									
Yu and Liang (2021)	DN	T:56(29) C:56(28)		T:60.85 ± 4.62 C:61.73 ± 6.58	KLX 2 g tid + Valsartan 80 mg qd	Valsartan 80 mg qd	8 w	②③④			

Notes: ①clinical symptoms; ②Scr; ③BUN; ④eGFR; ⑤ALB; ⑥UAER; ⑦24hUpro; ⑧mALB; ⑨ACR; ⑩FBG; ⑪postprandial blood glucose; ⑫HbA1c; ⑬TC; ⑭TG; ⑮LDL-C; ⑯HDL-C; ⑰adverse reactions; ⑱liver function; ⑲ECG; ⑳blood, urine, stool routine.

### 3.2 Risk of bias

**FIGURE 2:**
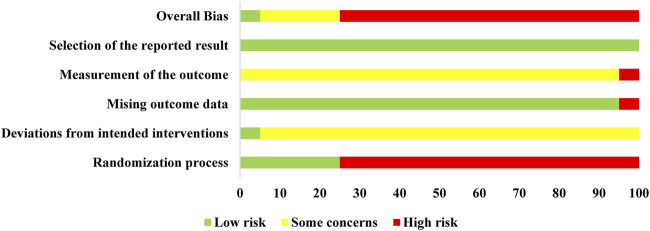
Risk of bias assessment in studies.

The risk of bias for the included trials is summarized in Figure 2 and Supplementary Figure S1. In the randomization process, there were five trials (Hou, 2012; Li et al., 2016; Zhang, 2016; Cui, 2021; Yu and Liang, 2021) that generated the allocation sequence by random number tables and one trial (Chen, 2014) that was randomized by patients' admission order, while the remaining trials only stated randomization without specific details of stochastic methods. None of the included trials described whether the allocation sequence was concealed. As for deviations from the intended intervention, only one trial performed a double-blind method. In the remaining trials, the participants were aware of their assigned interventions, but there was no information about the care or people delivering the interventions. Thus, there were some marked concerns. In terms of the outcome data, reports with complete results were judged to be low risk, except for one trial (Chen, 2014) without complete data that was judged to be high risk. On the measurement of the outcome, half of the included studies (He et al., 2012; Liu et al., 2012; Hu et al., 2013; Ju et al., 2013; Zhou, 2013; He et al., 2014; Shen, 2015; Hou, 2019; Jin, 2020; Yu and Liang, 2021) took biochemical indicators as outcomes and did not evaluate the effective rates; the remaining studies considered the specific symptoms and biochemical indicators as effective rates, so most were judged to have some concerns. All the outcomes were generated under a prespecified analysis plan and were considered low risk. Overall, only one trial (Zhang, 2016) was classified as low risk, which was conducted with a random numerical table method, double-blinding, placebo-controlled trial and four trials (Hou, 2012; Li et al., 2016; Cui, 2021; Yu and Liang, 2021) that were marked as having some concerns, while the remaining were judged as high risk. In conclusion, most of the involved clinical trials were deemed to be of poor methodological quality.

### 3.3 Meta-analysis results

#### 3.3.1 The primary outcomes

**FIGURE 3:**
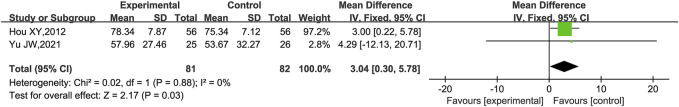
Forest plot of eGFR for KLXC + Western medicine vs. Western medicine alone (ml/min).

eGFR was reported in two trials (Hou, 2012; Yu and Liang, 2021). Heterogeneity analysis revealed no significant heterogeneity (*p* = 0.88, *I* ^2^ = 0%), and a fixed effect model was used for statistical analysis. The results showed that the group receiving KLXC combined with Western medicine was more likely to improve eGFR after 8 weeks of treatment than the group receiving Western medicine alone [MD = 3.04, 95% CI (0.30, 5.78), *p* = 0.03] (Figure 3).

**FIGURE 4:**
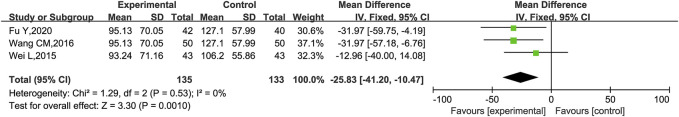
Forest plot of t mALB for KLXC + Western medicine vs. Western medicine alone (g/L).

mALB was reported in three trials (Wei et al., 2015; Wang, 2016; Fu et al., 2020). Heterogeneity analysis revealed no significant heterogeneity (*p* = 0.53, *I* ^2^ = 0%), and a fixed effect model was used for statistical analysis. The results showed that KLXC combined with Western medicine was significantly more effective in reducing mALB than when taking Western medicine alone [MD = −25.83, 95% CI (−41.20, −10.47), *p* = 0.001] (Figure 4).

**FIGURE 5:**
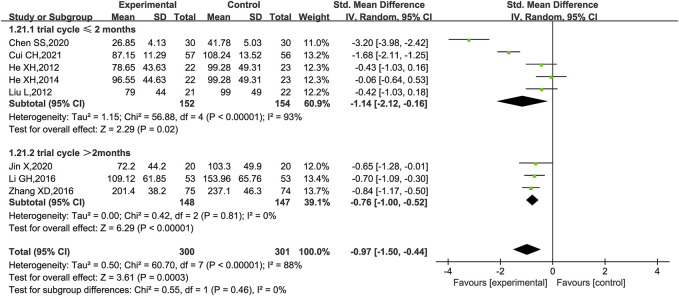
Forest plot of UAER for KLXC + Western medicine vs. Western medicine alone.

UAER was reported in nine trials (He et al., 2012; Liu et al., 2012; Chen, 2014; He et al., 2014; Li et al., 2016; Zhang, 2016; Chen et al., 2020; Jin, 2020; Cui, 2021), of which one trial (Chen, 2014) only stated that UAER was measured but had no data in the results, while the remaining eight trials were included for meta-analysis, of which five (He et al., 2012; Liu et al., 2012; He et al., 2014; Zhang, 2016; Jin, 2020) had UAER in mg/24 h and three trials (Li et al., 2016; Cui, 2021; Chen et al., 2020) had UAER in mg/min. Therefore, SMD was used to evaluate the difference. The result showed that KLXC combined with Western medicine was more effective in reducing UAER than when taking Western medicine alone [SMD = −0.97, 95% CI (−1.50, −0.44), *p* = 0.0003] (Figure 5). Heterogeneity analysis revealed highly significant heterogeneity (*p* < 0.00001, *I* ^2^ = 88%), thus a random effects model was used for statistical analysis, and a subgroup analysis was performed according to the trial cycle. The result showed that heterogeneity was significantly reduced in the group of trial cycle > 2 months (*p* = 081, *I* ^2^ = 0%), therefore the duration of therapy could be a source of high heterogeneity.

**FIGURE 6:**
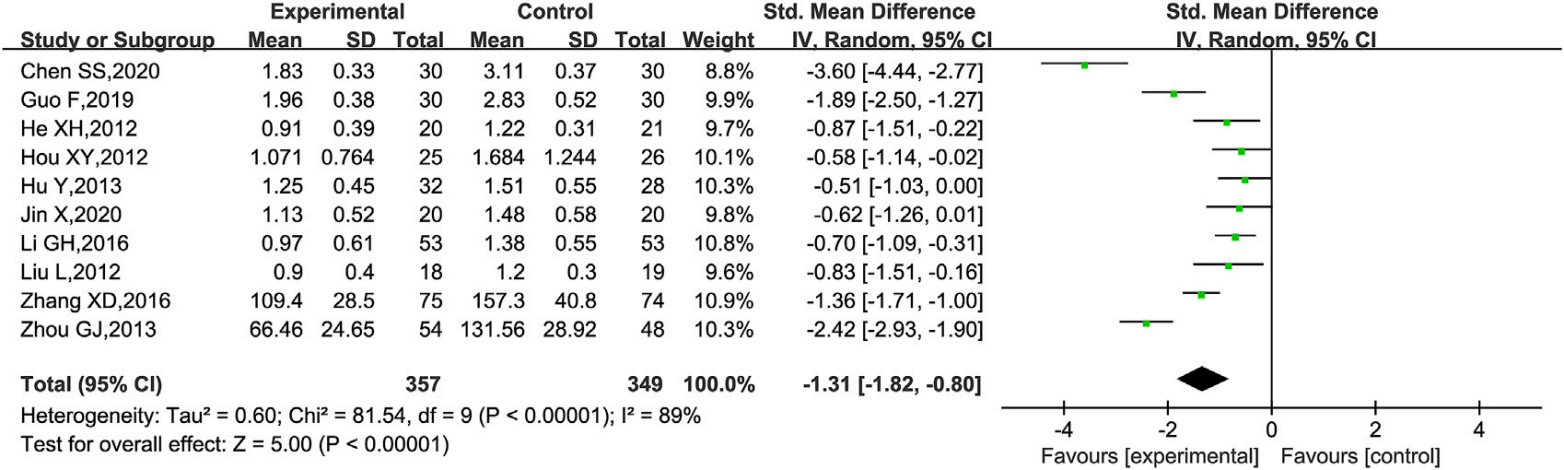
Forest plot of 24hUpro for KLXC + Western medicine vs. Western medicine alone.

24hUpro was reported in 10 trials (Hou, 2012; Liu et al., 2012; Hu et al., 2013; Zhou, 2013; He et al., 2012; Li et al., 2016; Zhang, 2016; Guo et al., 2019; Chen et al., 2020; Jin, 2020), of which 8 trials (Hou, 2012; Liu et al., 2012; Hu et al., 2013; He et al., 2014; Li et al., 2016; Guo et al., 2019; Chen et al., 2020; Jin, 2020) had 24hUpro unit in g and the remaining two (Zhou, 2013; Zhang, 2016) in mg. Thus, the forest plot was assessed by SMD. As shown in Figure 6, those who added KLXC in the experiment group had a significant advantage in reducing 24hUpro [SMD = −1.31, 95% CI (−1.82, −0.80), *p* < 0.00001], but the heterogeneity was high (*p* < 0.00001, *I* ^2^ = 89%). Thus, a random effects model was used for statistical analysis. However, in the search for the causes of high heterogeneity, neither subgroup analysis based on age and trial period grouping nor study-by-study deletion for sensitivity analysis can eliminate heterogeneity.

#### 3.3.2 The secondary outcomes

**FIGURE 7:**
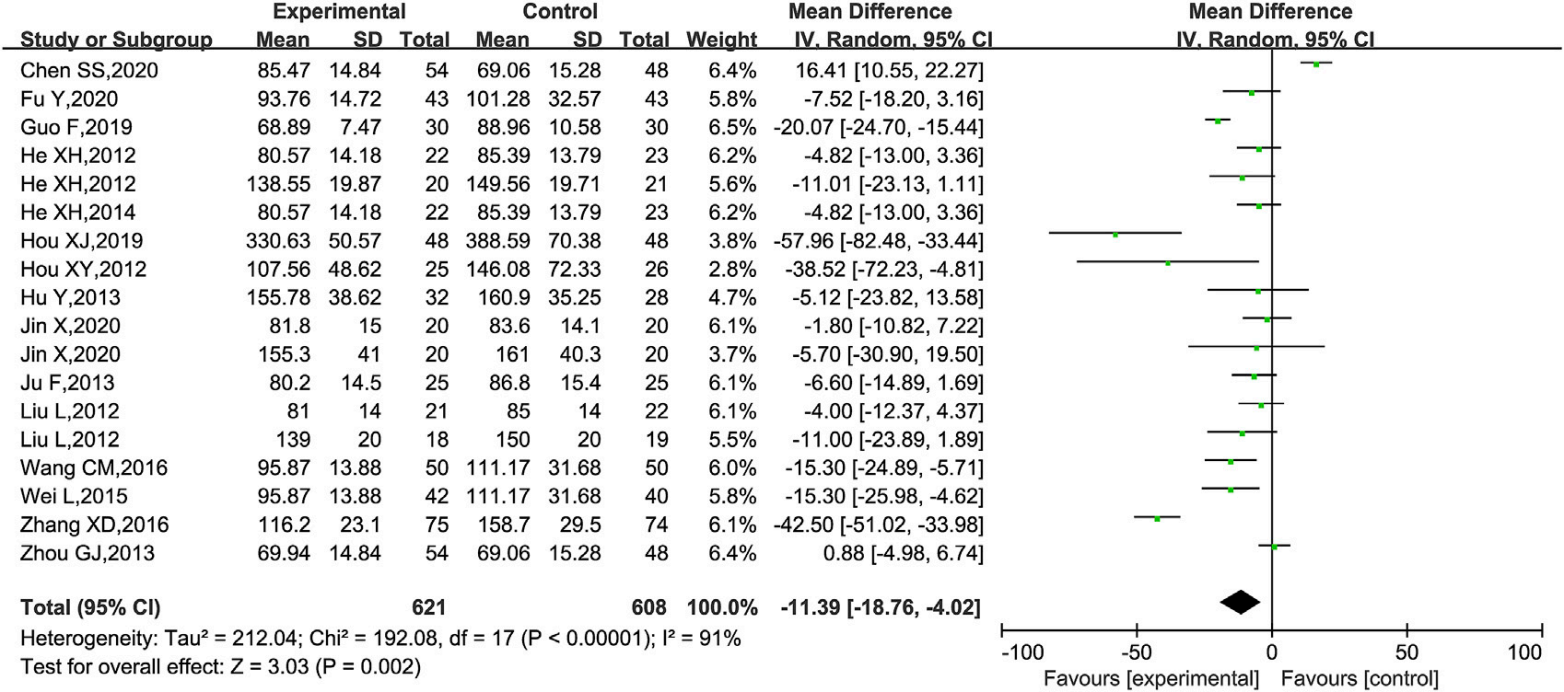
Forest plot of Scr for KLXC + Western medicine vs. Western medicine alone (μmol/L).

Scr was reported in 18 trials (He et al., 2012; Hou, 2012; Liu et al., 2012; Hu et al., 2013; Ju et al., 2013; Zhou, 2013; He et al., 2014; Shen, 2015; Wei et al., 2015; Wang, 2016; Zhang, 2016; Guo et al., 2019; Hou, 2019; Chen et al., 2020; Fu et al., 2020; Jin, 2020; Cui, 2021; Yu and Liang, 2021), of which 1 trial (Shen, 2015) was excluded as it only had a statistical *p*-value in the results and no specific data on Scr. The meta-analysis showed that KLXC combined with Western drug was more effective in reducing Scr than when taking Western drug therapy alone [MD = −11.39, 95% CI (−18.76, −4.02), *p* = 0.002] (Figure 7). Heterogeneity analysis revealed highly significant heterogeneity (*p* < 0.00001, *I* ^2^ = 91%), and a random effects model was used for statistical analysis. In the search for causes of high heterogeneity, heterogeneity remained at a high level, whether subgroup analyses were conducted by age, trial period, DKD stage grouping, or sensitivity analyses were performed by removing trials one by one.

**FIGURE 8:**
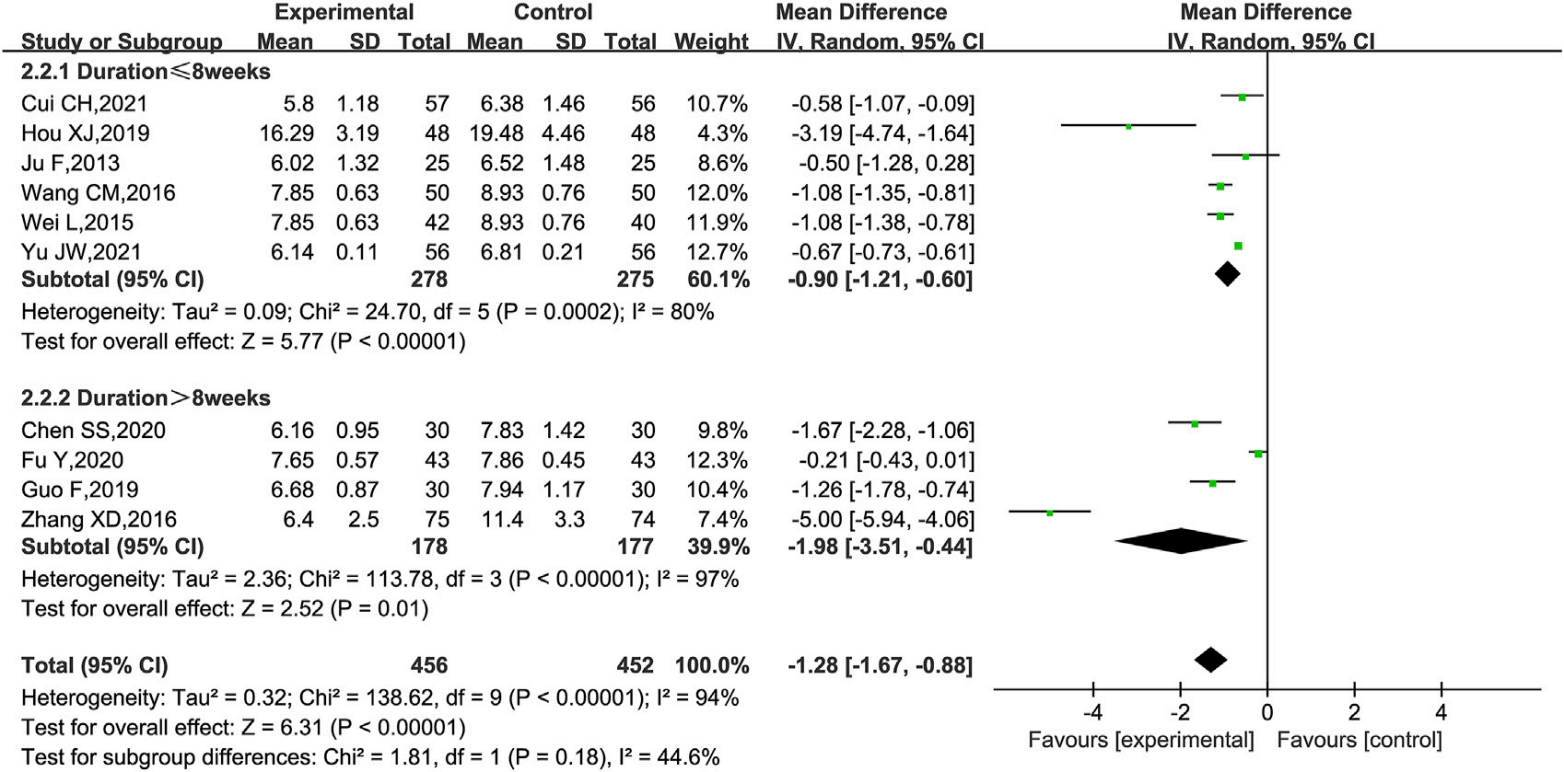
Forest plot of BUN for KLXC + Western medicine vs. Western medicine alone (mmol/L).

BUN was reported in 10 trials (Hou, 2012; Ju et al., 2013; Wei et al., 2015; Wang, 2016; Zhang, 2016; Guo et al., 2019; Chen et al., 2020; Fu et al., 2020; Cui, 2021; Yu and Liang, 2021). As shown in Figure 8, KLXC combined with Western medicine was more likely to reduce the level of BUN [MD = −1.28, 95% CI (−1.67, −0.88), *p* < 0.00001] than when taking Western medicine alone, but there was significant heterogeneity between the studies (*p* < 0.00001, *I* ^2^ = 94%); a random effects model was used for statistical analysis. When the subgroup analyses were performed according to the trial cycle or stage of DKD, there remained high heterogeneity.

**FIGURE 9:**
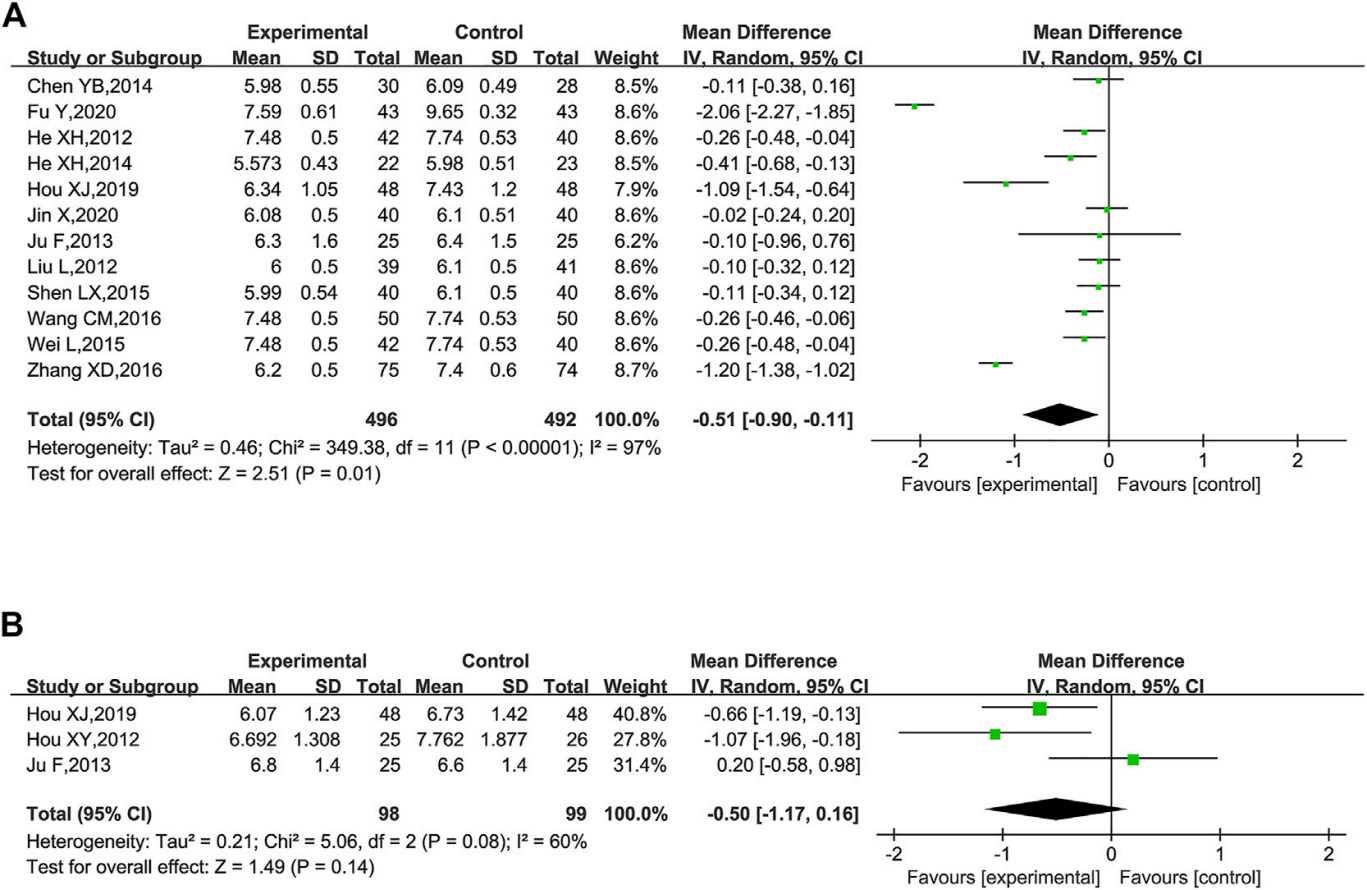
Forest plot of glucose for KLXC + Western medicine vs. Western medicine alone. **(A)** Forest plot of FBG for KLXC + Western medicine vs. Western medicine alone. **(B)** Forest plot of HbA1c for KLXC + Western medicine vs. Western medicine alone.

FBG was reported in 12 trials (He et al., 2012; Liu et al., 2012; Ju et al., 2013; Chen, 2014; He et al., 2014; Shen, 2015; Wei et al., 2015; Wang, 2016; Zhang, 2016; Hou, 2019; Fu et al., 2020; Jin, 2020). These studies plus KLXC with Western medicine showed a slight decrease in FBG when compared to taking Western medicine alone [MD = −0.51, 95% CI (−0.90, −0.11), *p* = 0.01], but with significant heterogeneity between the studies (*p* < 0.00001, *I* ^2^ = 97%) (Figure 9A). Also, heterogeneity remained high when further subgroup analyses were performed for age and glucose grouping.

HbA1c was reported in three trials (Hou, 2012; Ju et al., 2013; Hou, 2019). The forest plot illustrated no statistically significant difference in HbA1c between the two groups (*p* = 0.14), and the heterogeneity test suggested a high degree of heterogeneity (*p* = 0.08, *I* ^2^ = 60%) (Figure 9B). When each study was removed in turn for sensitivity analysis, similar heterogeneity remained.

**FIGURE 10:**
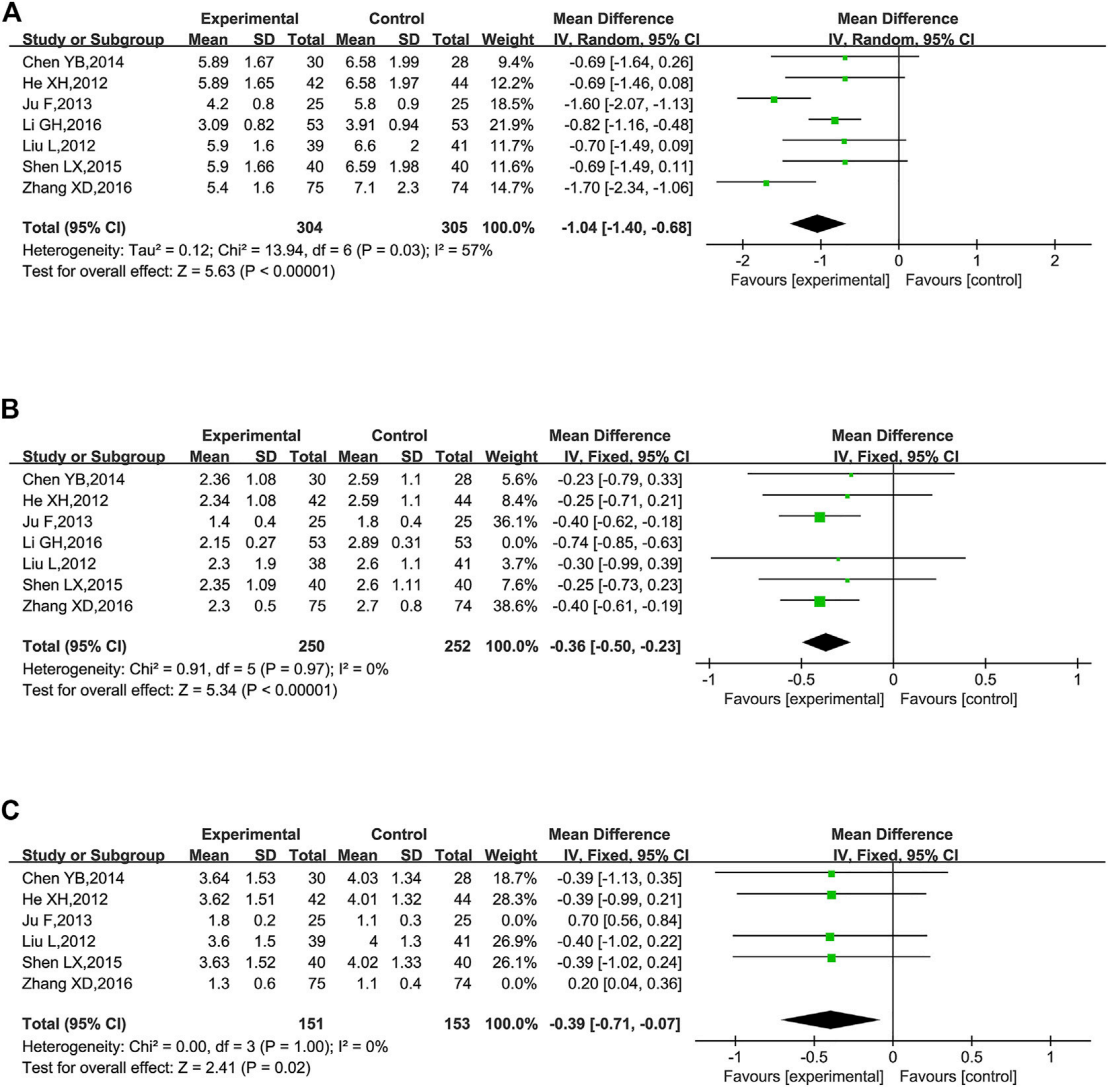
Forest plot of lipids for KLXC + Western medicine vs. Western medicine alone (mmol/L). **(A)** Forest plot of TC for KLXC + Western medicine vs. Western medicine alone. **(B)** Forest plot of TG for KLXC + Western medicine vs. Western medicine alone. **(C)** Forest plot of LDL for KLXC + Western medicine vs. Western medicine alone.

TC were reported in seven trials (He et al., 2012; Liu et al., 2012; Ju et al., 2013; Chen, 2014; Shen, 2015; Li et al., 2016; Zhang, 2016). The combination of KLXC with Western medicine significantly reduced the TC levels when compared to taking Western medicine alone [MD = −1.04, 95% CI (−1.40, −0.68), *p* < 0.00001], but there was slightly higher heterogeneity between the two studies (*p* = 0.03, *I* ^2^ = 57%) (Figure 10A).

TG was reported in seven trials (He et al., 2012; Liu et al., 2012; Ju et al., 2013; Chen, 2014; Shen, 2015; Li et al., 2016; Zhang, 2016). The heterogeneity test suggested a high degree of heterogeneity (*p* < 0.00001, *I* ^2^ = 91%), and sensitivity analysis was carried out by excluding trials one by one. Heterogeneity was significantly reduced after removing the study reported by Li et al. (2016) (*p* = 0.97, *I* ^2^ = 0%). As shown in Table 2, different from the other 6 trials, Li’s research did not explicitly report the stage of DKD, which might contribute to high heterogeneity. A fixed effects model was used for meta-analysis after removing Li’s report. The result showed that the combination of KLXC with Western medicine was more effective in reducing the TG levels than when taking Western medicine alone [MD = −0.36, 95% CI (−0.50, −0.23), *p* < 0.00001] (Figure 10B).

LDL was reported in six trials (He et al., 2012; Liu et al., 2012; Ju et al., 2013; Chen, 2014; Shen, 2015; Zhang, 2016). Significant heterogeneity was detected among these studies (*p* < 0.00001, *I* ^2^ = 89%). When sensitivity analysis was performed by deleting the studies one by one, heterogeneity remained high. When the two reported studies of Zhang (2016) and Ju et al. (2013) (*p* = 1.00, *I* ^2^ = 0%) were removed simultaneously, the heterogeneity was significantly reduced and we found that both studies met the lowest threshold of LDL for atherosclerotic cardiovascular disease (Grundy et al., 2019), and the remaining four trials had hypercholesterolemia of LDL. Thus, the level of LDL might be the main source of high heterogeneity. A fixed effect model was used for meta-analysis after moving the two studies (Ju et al., 2013; Zhang, 2016). The result showed that KLXC combined with Western medicine was more effective in reducing LDL than taking Western medicine alone [MD = −0.39, 95% CI (−0.71, −0.07), *p* = 0.02] (Figure 10C).

#### 3.3.3 Other outcomes

**FIGURE 11:**
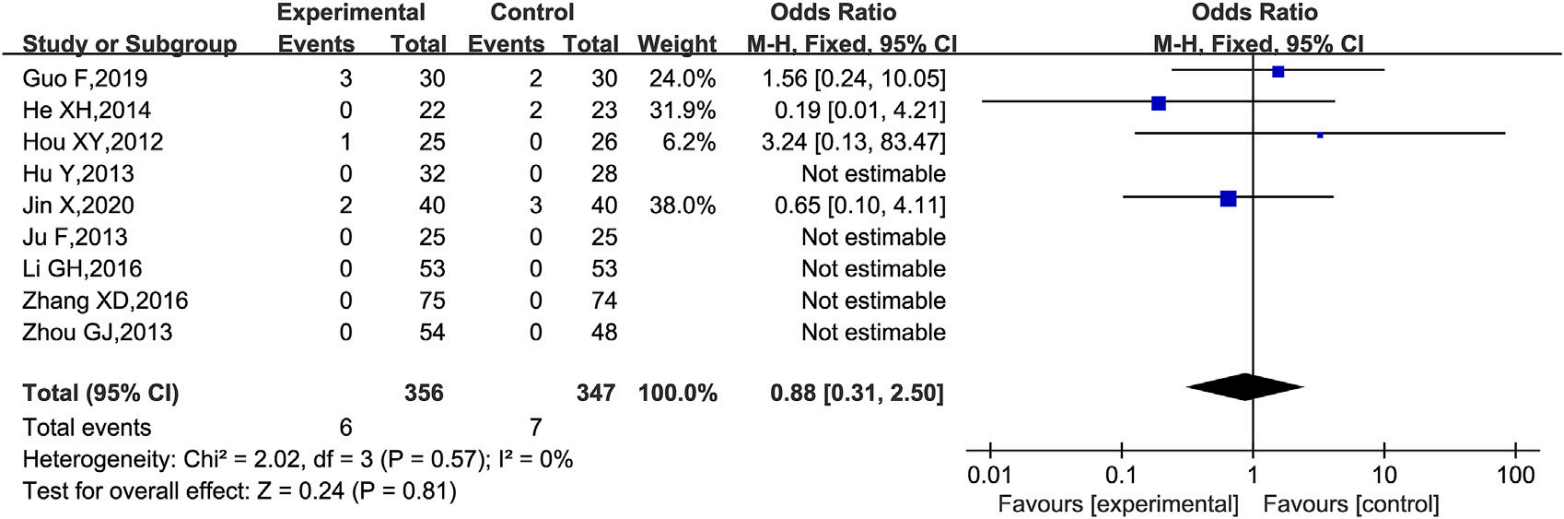
Forest plot of adverse events for KLXC + Western medicine vs. Western medicine alone.

Adverse events were reported in 10 trials (Hou, 2012; Hu et al., 2013; Ju et al., 2013; Zhou, 2013; Chen, 2014; He et al., 2014; Li et al., 2016; Zhang, 2016; Guo et al., 2019; Jin, 2020), of which one trial (Chen, 2014) was excluded because the adverse reactions were planned to be assessed in the protocol but not reported in the results, and the remaining nine trials (Hou, 2012; Hu et al., 2013; Ju et al., 2013; Zhou, 2013; He et al., 2014; Li et al., 2016; Zhang, 2016; Guo et al., 2019; Jin, 2020) were analyzed for adverse reactions. There were two cases of diarrhea (Hou, 2012; Jin, 2020), two cases of nausea and vomiting (Guo et al., 2019; Jin, 2020), one case of skin rash (Lin et al., 2000), and one case of hypercalcemia (Guo et al., 2019) in the group with the combination of KLXC and Western medicine. There were two cases of abdominal irritation (He et al., 2014), two cases of nausea and vomiting (He et al., 2012; Hou, 2012; Hu et al., 2013; He et al., 2014; Guo et al., 2019; Hou, 2019; Jin, 2020), two cases of hypercalcemia (Guo et al., 2019), and one case of cough (Jin, 2020) in the control group treated with Western medicine alone. Five trials (Hou, 2012; Hu et al., 2013; Ju et al., 2013; Zhou, 2013; Zhang, 2016) monitored liver function before and after the intervention, and none reported abnormal liver functions. All adverse effects disappeared after appropriate treatment and continued medication, except for one case in the experiment group that was discontinued due to diarrhea (Hou, 2012). According to the forest plot results, the OR for the adverse events was 0.88 and the 95% CI for the OR was [0.31, 2.50] which included 1.00, implying that the OR was not statistically significant. Thus, there was no statistical difference in the adverse events between the experiment group (KLXC + Western medicine) and control group (Western medicine alone) [OR 0.88 (0.31, 2.50), *p* = 0.81]. The heterogeneity analysis revealed no significant heterogeneity (*p* = 0.57, *I* ^2^ = 0%) (Figure 11).

#### 3.3.4 Publication bias assessment

**FIGURE 12:**
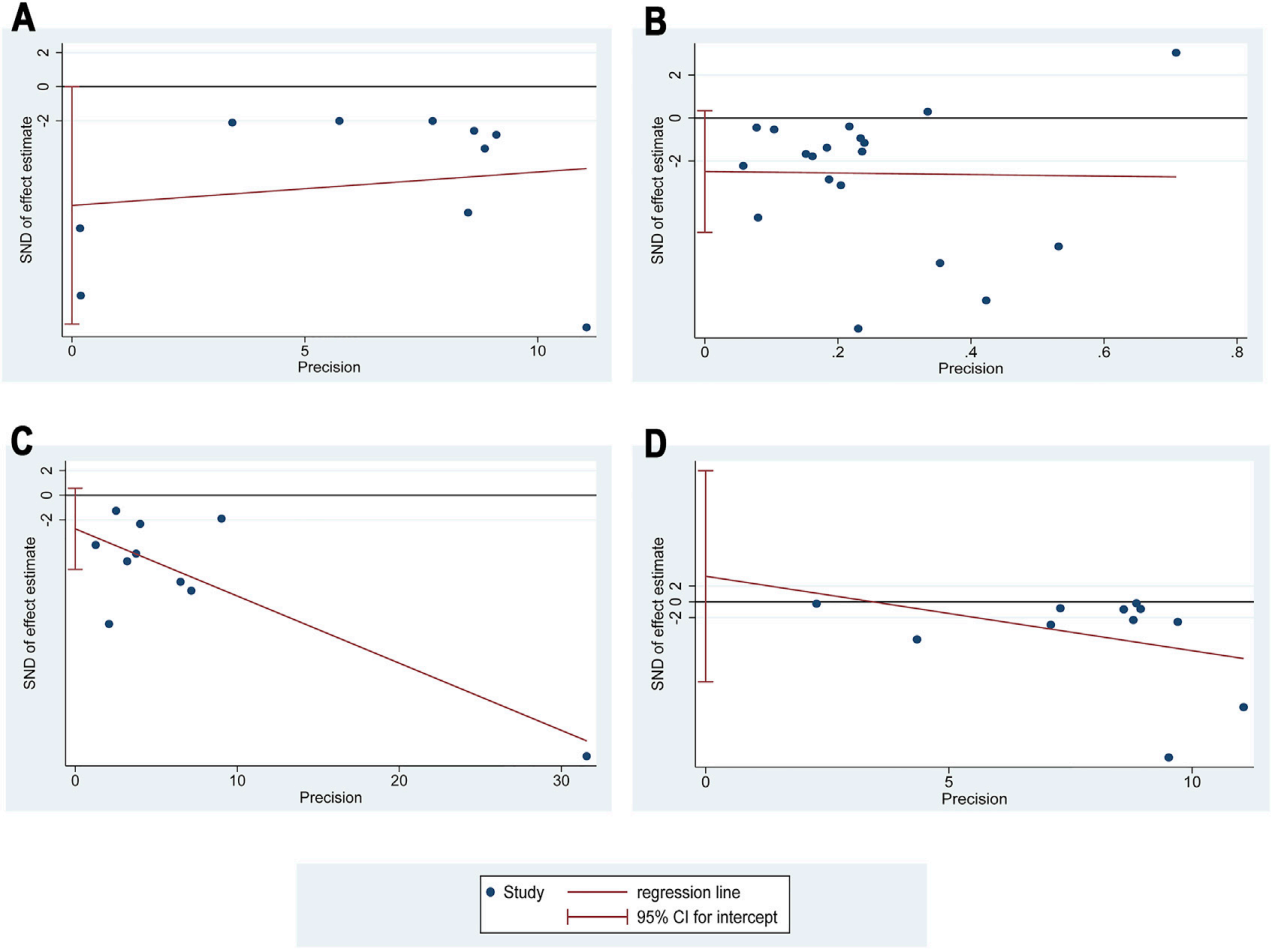
Egger’s publication funnel plot. **(A)** Egger’s funnel plot of 24hUpro. **(B)** Egger’s funnel plot of Scr. **(C)** Egger’s funnel plot of BUN. **(D)** Egger’s funnel plot of FBG.

At least 10 studies reported 24hUpro (10 RCTs), Scr (18 RCTs), BUN (10 RCTs), and FBG (10 RCTs). So these four outcomes were selected for Egger’s test assessment to assess publication bias by Stata 17.0 (Figure 12). The results showed that the *p*-values were 0.05 (24hUpro), 0.081 (Scr), 0.092 (BUN), 0.6 (FBG), and these *p*-values were not less than 0.05, indicating that there was no significant publication bias among the studies.

## 4 Discussion

### 4.1 Summary of evidence

The coexistence of multiple risk factors and emergence of multiple complications in DKD increased the complexity of management and the diversity of medications (Jiang et al., 2020). It is increasingly emphasized that simultaneous control of blood pressure, glucose, and lipids can help maximize the benefits for DKD patients (Ueki et al., 2021). Therefore, there is an urgent need to comprehensively evaluate the efficacy and safety of medications with multiple therapeutic effects through high-quality evidence-based medicine. In this review, we systematically analyzed the 20 included RCTs that compared the effects of the addition of KLXC on renal functions, glucose, and lipid levels in 1,500 DKD patients. This analysis revealed a significant advantage of the combination of KLXC and Western medicine on renal functions (eGFR, mALB, UAER, 24hUpro, Scr, and BUN), FBG, and lipids (TC, TG, and LDL). In addition, the combination therapy also demonstrated better efficacy in the clinical efficiency rate involving both clinical symptoms and biochemical indicators. Thus, KLXC exhibited significant comprehensive adjuvant treatment advantages for DKD.

The natural progression of DKD was described as progressive albuminuria followed by a decline in eGFR (Tuttle et al., 1990). With the intensive management of DKD, which included intensive glucose and lipid control, improvement in blood pressure, and the use of drugs such as ARBs, ACEI, and SGLT2, the prevalence of albuminuria decreased. However, the increasing incidence of reduced eGFR (Afkarian et al., 1988-2014) and the mechanism still remains unclear and may be related to retinopathy and macrovascular disease (de Boer et al., 2011). In this systematic review, we found that the addition of KLXC reduced both proteinuria (mALB, UAER, and 24hUpro) and the improved eGFR when compared to taking Western drugs alone, which might be the therapeutic advantage of KLXC. However, there was high heterogeneity in UAER and 24hUpro. The trial cycle may be the main heterogeneity of UAER, however, we failed to find the source of heterogeneity in 24hUpro. Recent mechanistic studies have shown that KLXC not only protected the glomerulus but also inhibited the tissue inhibitor of matrix metalloproteinase-1 (TIMP-1) overexpression, increased matrix metalloprotein-9 (MMP-9) expression, and protected tubular basement membrane in rats. It was also associated with protein kinase R–like ER kinase–activating transcription factor 4–CCAAT/enhancer-binding protein homologous protein (PEPK-ATF4-CHOP) pathway–mediated apoptosis of renal tubular epithelial cells and endoplasmic reticulum stress (Wei, 2022). Meanwhile, the constituent Chinese medicines in KLXC also proved to be beneficial for DKD. The animal experiments showed that the active ingredients of *A. membranaceus* were effective in reducing FBG and albuminuria levels, reversing the glomerular hyperfiltration state, and ameliorating the pathological changes of early DKD in rat models (Zhang et al., 2009) and network pharmacology revealed that the targets of *A. membranaceus* for DKD were related to some biological process such as inflammatory response, angiogenesis, and oxidative stress reaction (Guo et al., 2020). Moreover, a meta-analysis revealed that *A. membranaceus* preparations were effective and tolerable for the short-term reduction of albuminuria and Scr in DKD patients (Zhang et al., 2019). Animal experiments showed that hirudin, a natural compound of the leech, inhibited inflammation and podocyte apoptosis in DKD rats *via* the p38 mitogen–activated protein kinase/nuclear factor κB (MAPK/NF-κB) pathway (Han et al., 2020). Rheum officinale reduced glucose and lipids and inhibited oxidative stress in diabetic rats, thereby alleviating nephrotic damage (Hosseini et al., 2017).

DKD frequently coexisted with multiple metabolic disorders. In addition to hyperglycemia, other factors such as hypertension, dyslipidemia, and an unhealthy lifestyle were related to the pathogenesis of DKD (Jiang et al., 2020). It is widely known that hyperglycemia is a primary persistent driver of DKD and intensive glycemia undoubtedly helps control the progression of DKD. We found that KLXC decreased the level of FBG but had no significant reduction of HbA1c which reflect the 120-day average of glucose rather than the point value. The HbA1c may be influenced by the erythrocyte renewal cycle, hemoglobin concentration, acid–base balance, and medicines, which are the main reasons for the inaccuracy of HbA1c (Ansari et al., 2003). Meanwhile, the source for the high heterogeneity of FBG and HbA1c has not been found in this study because of inadequate sample and documentation.

A meta-analysis revealed that high TG levels were an independent risk factor for DKD over 4 years (Russo et al., 2016). Lipotoxicity caused by lipid abnormalities and renal lipid accumulation proved to be associated with damage to the tubule and glomerulus, which promoted kidney dysfunction (Opazo-Ríos et al., 2020). Meanwhile, insulin resistance caused by dyslipidemia also contributed to kidney dysfunction by inflammatory or podocyte damage, which promoted glomerular hyperfiltration and vascular permeability (De Cosmo et al., 2013; Artunc et al., 2016). In this meta-analysis, the combination of KLXC and Western medicine significantly decreased the levels of TC, TG, and LDL more than when taking Western medicine alone. We judged that the higher heterogeneity may be caused by the duration of intervention and the baseline of the lipids.

Thus, KLXC combined with Western medicine showed a significant advantage of the comprehensive therapy for DKD. However, there were some heterogeneities in the glucolipid metabolism outcomes which might be related to various factors such as the stage of DKD, basal values of lipids and glucose, and trial period. Therefore, the factors that affected the results and inclusion of a strict baseline for specific indicators should be refined in future RCTs.

Apart from efficacy, the safety of KLXC should be an equally important consideration, which not only helps find ways to alleviate adverse effects but also guarantees the safety of the drug. However, only half of the included RCTs recorded specific adverse reactions and the corresponding numbers of which five RCTs monitored liver function (Table 2). Based on the adverse reactions reported in the 10 trials, a total of 4 patients in the experiment group experienced gastrointestinal discomfort, which may be related to the greater stimulation of the animal drug or laxative effect of Rheum officinale (Wang et al., 2012). One patient in the experiment group suffered from hypercalcemia, and further research is still required to reveal the possible mechanism. Also, one patient presented with skin rash, which may also be related to drug-induced immune reaction. Although these symptoms were alleviated or disappeared after drug reduction, discontinuation, or symptomatic treatment, there is still a great demand for comprehensive monitoring of side effects and detailed documentation in reports, which includes but is not limited to the symptoms, liver and kidney functions, ECG, and other physical signs. This will help improve the awareness of adverse drug reactions and stimulate research on the mechanisms of adverse reactions.

### 4.2 Strengths and limitations

This is the first systematic review and meta-analysis of KLXC for DKD in English. Compared with a similar meta-analysis published in China in 2019^[10]^, our study updated four RCTs from 2019 onwards and added the systematic evaluation of eGFR, mALB, UAER, and HbA1c. Bai’s research reported no significant difference between group comparisons in 24hUpro, but in our research, the combination of KLXC and Western medicine showed better efficacy than taking Western medicine alone as the sample increased. In addition, we performed subgroup and sensitivity analyses to analyze the high heterogeneity of the outcomes and prospected some implications for future studies on KLXC.

However, our results might be limited by the following aspects. Firstly, most of the included trials were not standard RCTs and were more biased toward real-world studies, and only one trial was randomized and double blinded with high evidence support. Although randomizations were shown in the trials, none of them described the specific randomization method and the concealment of the randomization sequence was a serious problem in many studies. Secondly, none of the studies included in this review had follow-up at the end of the intervention, which was not conducive in observing long-term time effects and dose effects. Thirdly, all the included trials were conducted and published in China, and conclusions may not exclude bias due to geographical and ethnic influences.

### 4.3 Implication

In addition, the greater benefit of this article might be awareness of current problems and areas for further improvement. 1) Clinical trials that include randomization, allocation concealment, and blinding should be strictly designed and described in detail in the report. 2) The stage of DKD included in the study should be clarified. 3) The assessment of clinical effective rates should be comprehensive and should include a composite of symptoms, blood pressure, renal function, glucose, lipids, and other indicators. 4) Long-term follow-up after the trials are also necessary, especially for end-point events.

## 5 Conclusion

In conclusion, the systematic review and meta-analysis suggest that KLXC combined with Western medicine is superior to taking Western medicine alone during conventional therapy. However, due to the high clinical heterogeneity and unstandardized nature of the included trials, large-scale, randomized, double-blind, multicenter RCTs at different stages of DKD are required to evaluate or confirm the current results.

## Data Availability

The original contributions presented in the study are included in the article/Supplementary Material; further inquiries can be directed to the corresponding author.
