# Supplementary material for: Efficacy and safety of Keluoxin capsule in combination with Western medicine for diabetic kidney disease: A systematic review and meta-analysis
Source: Front Pharmacol. 2023 Jan 4;13:1052852. doi: 10.3389/fphar.2022.1052852 (PMC9845565; doi:10.3389/fphar.2022.1052852)
Supplement: Supplementary file 1 [file Table1.DOCX]

Supplementary Table 1| The search queries for each database.

| Database | Search Queries |
| --- | --- |
| CNKI | SU %= 'Keluoxin' AND SU %= ' Diabetic Kidney Disease ' AND FT = ' Random' |
| WF data | Topic:( Keluoxin) and Topic:( Diabetic Kidney Disease) and All:( Random) |
| VIP | M=Keluoxin AND M=Diabetic Kidney Disease AND U=Random |
| SinoMED | " Keluoxin "[ All fields: Auto] AND " Diabetic Kidney Disease "[ All fields: Auto] AND " Random "[ All fields: Auto] |
| PubMed | ((Keluoxin) AND ((Diabetic Kidney Disease) OR (Diabetic Nephropathy))) AND (Random) |
| WOS | ALL=(Keluoxin) AND ((ALL= (Diabetic Kidney Disease)) OR ALL=(Diabetic Nephropathy)) AND ALL=(Random) |
| CLib | (Keluoxin): ti,ab,kw AND (Diabetic Kidney Disease):ti,ab,kw AND (random) |
