# Supplementary material for: Efficacy and safety of Keluoxin capsule in combination with Western medicine for diabetic kidney disease: A systematic review and meta-analysis
Source: Front Pharmacol. 2023 Jan 4;13:1052852. doi: 10.3389/fphar.2022.1052852 (PMC9845565; doi:10.3389/fphar.2022.1052852)
Supplement: Supplementary file 2 [file Image1.pdf]

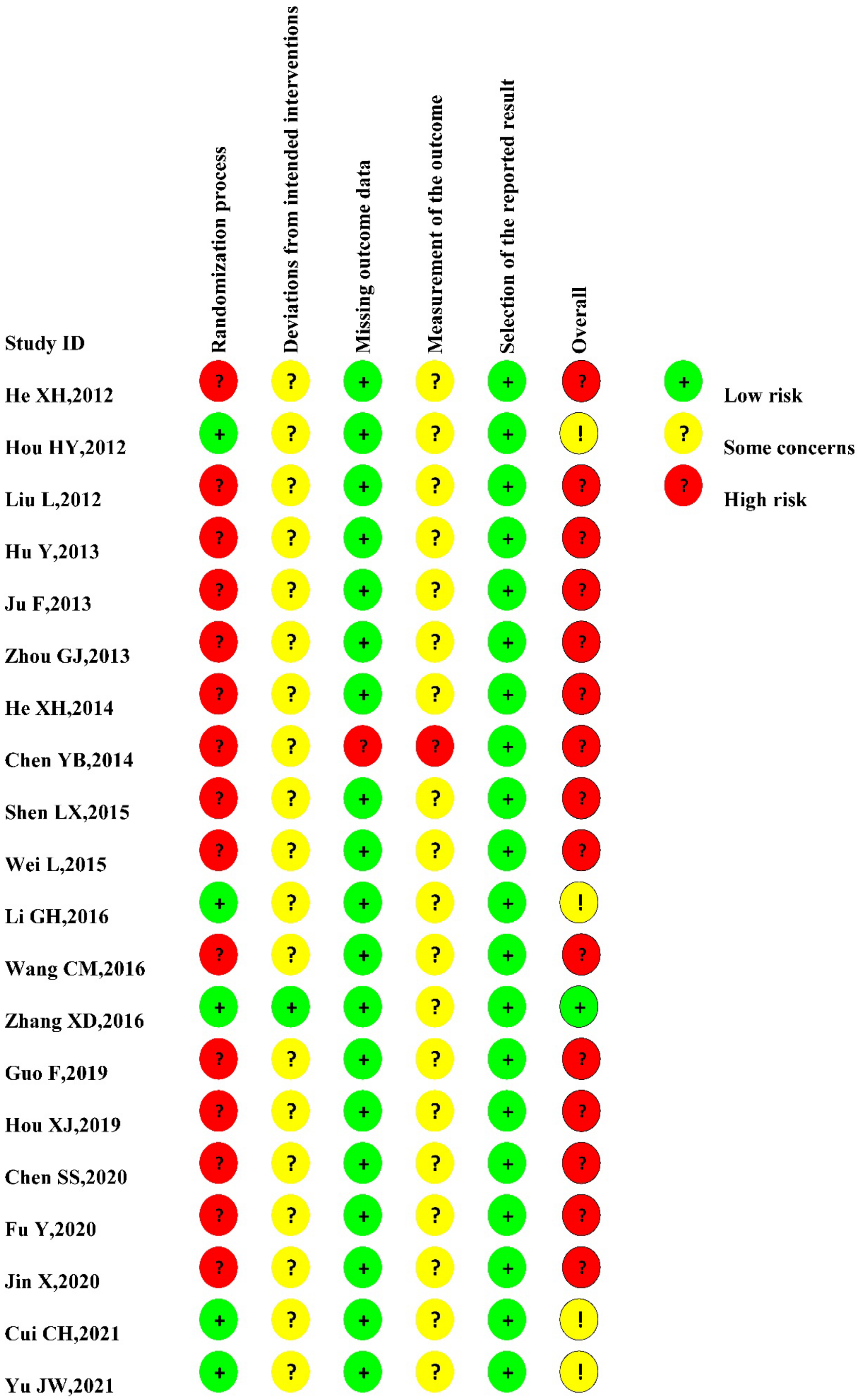

Supplementary Figure 1 | Risk of bias assessment for each included study in the

review. Each row represents a study, each column represents an aspect of the evaluation, and the last column represents the overall evaluation of this study. The green circles represent low risk, the yellow circles represent controversial evaluations, and the red circles represent high risk.
